# Cornual Pregnancy as a Rare Entity of Ectopic Pregnancy After Assisted Reproductive Therapy‐Embryo Transfer (ART‐ET): A Case Report

**DOI:** 10.1002/ccr3.9708

**Published:** 2024-12-06

**Authors:** Fatemeh Keikha, Hawraa Shbeeb, Huma Homam, Najmeh Nasiri Khormoji, Mahdiye Nouri

**Affiliations:** ^1^ Department of Obstetrics and Gynecology Family Health Research Institute, Imam Khomeini Hospital Complex, Vali‐e‐Asr Hospital, Tehran University of Medical Sciences Tehran Iran

**Keywords:** cornual pregnancy, embryo transfer, in vitro fertilization (IVF), salpingectomy

## Abstract

Cornual pregnancy, a rare and life‐threatening form of ectopic pregnancy, poses significant diagnostic and therapeutic challenges due to its deep implantation in the uterus. This report presents a case of a 31‐year‐old woman with a history of assisted reproductive technology (ART) and prior salpingectomy, who was diagnosed with a right cornual pregnancy following embryo transfer. The patient experienced lower abdominal pain and was found to have an enlarged uterus on ultrasonography. Early diagnosis via three‐dimensional transvaginal ultrasonography enabled timely intervention, preventing rupture and severe hemorrhage. Surgical management involved a combined hysteroscopy and minilaparotomy to resect the cornual pregnancy. Histopathology confirmed the diagnosis, and the patient's recovery was uneventful. Elective cesarean section at 36–37 weeks was advised for future pregnancies to prevent uterine rupture.

**Summary**

- Ectopic pregnancies, especially cornual implantations after IVF‐ET, require vigilant monitoring due to the risk of early rupture and severe hemorrhage.
- Prior salpingectomy does not eliminate this risk; therefore, posing management challenges.
- Careful follow‐up with transvaginal ultrasound, serial HCG measurements is essential for detection after IVF‐ET.

## 1 Introduction

Cornual pregnancy, a rare subtype of ectopic pregnancy, occurs at the cornual or interstitial part of the uterus where the fallopian tube intersects with the uterine cavity. Representing 2%–4% of all ectopic pregnancies, these cases pose significant challenges due to their potential for severe hemorrhage and high mortality rates, especially when diagnosis is delayed until rupture [1]. Risk factors increasing the likelihood of cornual pregnancies include impaired tubal function from prior inflammatory diseases, surgical interventions such as salpingectomy, and the increasing use of assisted reproductive technologies (ART) [1, 2]. These pregnancies are particularly prone to misdiagnosis and subsequent complications due to their deep implantation, which allows significant growth before detection, hence increasing the risk of rupture and severe hemorrhage [1, 2]. The management of such cases often necessitates vigilant monitoring, timely surgical intervention, and prompt treatment to prevent adverse outcomes [1, 2]. Any subsequent pregnancies following a cornual pregnancy are generally managed through elective cesarean section around 36 to 37 weeks to prevent uterine rupture [3].

## 2 Case Presentation

### 2.1 Case History/Examination

A 31‐year‐old woman, gravida 2, abortus 1, presented to the emergency department (ED) with lower abdominal pain after undergoing an embryo transfer procedure. She reported a history of primary infertility for 4 years and secondary infertility for 1 year. She had one spontaneous abortion at 10 weeks in the previous year without a history of curettage. The patient had undergone a laparoscopic salpingectomy due to hydrosalpinx last year, although it was unclear whether the procedure was bilateral or unilateral. Additionally, she had a laparotomy for endometriosis via a Pfannenstiel incision 5 years ago.

She was 5 weeks and 6 days pregnant after an embryo transfer involving two frozen embryos with one successful implantation. The pregnancy was initially confirmed through serial beta‐HCG measurements, which showed appropriate rises consistent with early gestation. As per routine protocol, a transvaginal ultrasound was performed.

The patient's medications included daily 1 mg folic acid, 1000 U Vitamin D3, and 80 mg Aspirin. Following an IVF‐ET procedure, she was on a treatment plan consisting of 6 mg oral estradiol and 400 mg progesterone suppositories administered vaginally twice per day for luteal phase support.

### 2.2 Methods (Differential Diagnosis, Investigations, and Treatment)

Her physical examination showed stable vital signs and mild pallor, with no signs of peritoneal irritation. Pelvic examination showed a tender and enlarged uterus, there was no cervical motion tenderness or vaginal bleeding; however, a brownish vaginal discharge was present.

**FIGURE 1:**
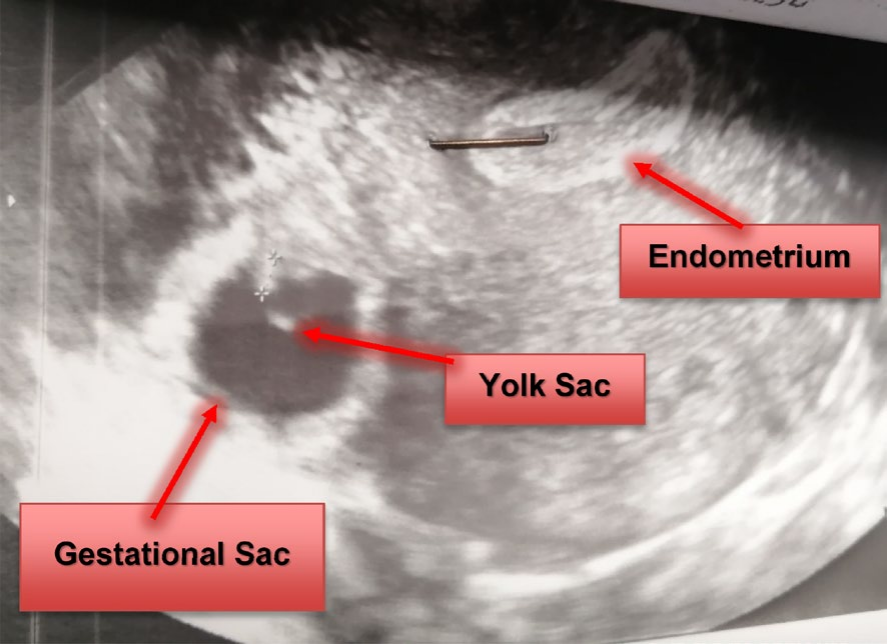
A transvaginal ultrasound revealed a right cornual ectopic pregnancy with embryo and yolk sac and the uterine cavity with no intrauterine pregnancy.

**FIGURE 2:**
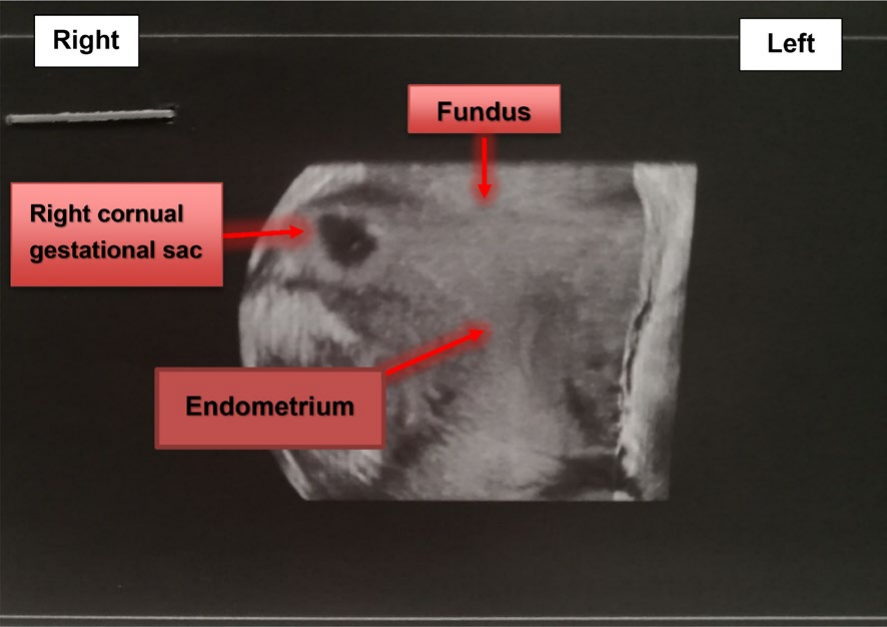
Three‐dimensional transvaginal ultrasound revealed that the gestational sac was not connected with endometrium.

The initial transvaginal ultrasonography raised suspicions of a right cornual pregnancy with an endometrial thickness of 18 mm, and no signs of intrauterine pregnancy. No free fluid was detected in the cul‐de‐sac (Figure 1). Serum beta‐subunit of human chorionic gonadotrophin (Beta‐HCG) level was 2827 IU/mL on the 14th day post‐embryo transfer and had increased to 22,628 IU/mL at the time of hospital admission. The patient's hemoglobin level was 12.8 g/dL, and her total white cell count was 9 g/L. Coagulation tests (PTT/PT/INR) were within normal limits. A subsequent detailed three‐dimensional transvaginal ultrasound (3D) confirmed the presence of a gestational sac, measuring 94 × 46 mm with a Crown‐Rump Length (CRL) of 2.7 mm showing cardiac activity, embedded into the right cornual wall and disconnected from the uterine cavity (Figure 2).

**FIGURE 3:**
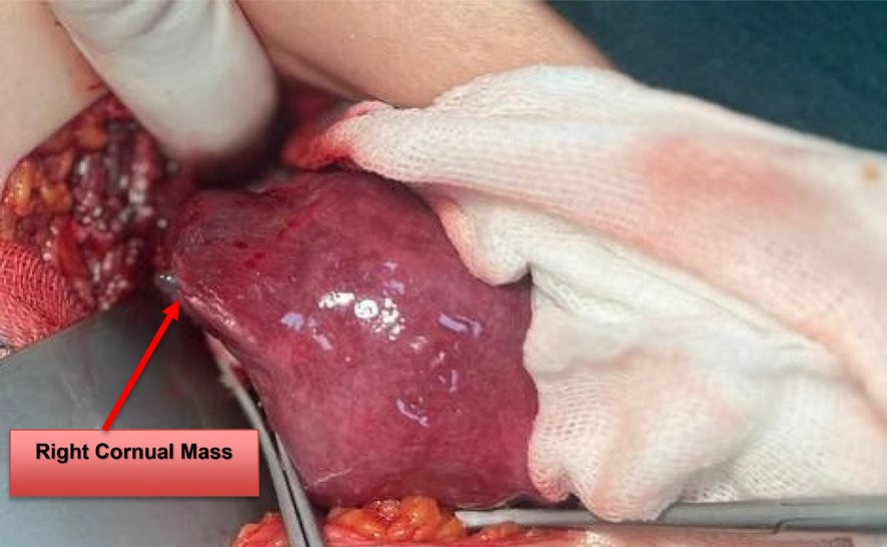
Minilaparotomy revealing a right intact cornual mass.

Informed consent was obtained for a combined hysteroscopy and minilaparotomy under general anesthesia. Diagnostic hysteroscopy revealed a decidualized endometrium without evidence of intrauterine pregnancy, and both uterotubal ostia were visualized normally (Figure 3). The cornual pregnancy was not visible or accessible during the hysteroscopic examination. Due to the very thin myometrial layer (< 5 mm) surrounding the gestational sac and the risk of rupture from increased intrauterine pressure, the surgical team decided not to proceed further to avoid the risk of massive intra‐abdominal bleeding. Therefore, we decided to perform a minilaparotomy.

A minilaparotomy confirmed an enlarged uterus and a 4 × 2.5 cm mass located in the right cornual region, covered only by serosa and a very thin layer of the myometrium, on the verge of potential rupture without active bleeding or rupture (Figure 3). Both ovaries and the left fallopian tube appeared normal, but the right fallopian tube was absent due to a previous salpingectomy. After administering dilute vasopressin (20 units in 100 mL of injectable saline) into the myometrium surrounding the pregnancy, a right cornual resection was performed, and hemostasis was achieved. The pedicle was sutured with 1‐Vicryl, and the specimen was sent for histopathological examination.

### 2.3 Conclusion and Results (Outcome and Follow‐Up)

Histopathological examination confirmed the diagnosis of a right cornual pregnancy, identifying products of conception and chorionic villi. The patient's recovery was uneventful, and she was discharged in stable condition 2 days post‐surgery, receiving antibiotic coverage and NSAIDs. For her future pregnancies, an elective cesarean section at 36–37 weeks was advised to prevent uterine rupture.

## 3 Discussion

Cornual pregnancies, a rare ectopic pregnancy subtype, present significant diagnostic and therapeutic challenges due to their deep implantation within the uterine wall. The presented case mirrors the literature regarding diagnostic complexities and the need for early intervention to prevent rupture and severe hemorrhage [1, 3].

Cornual pregnancies often grow undetected until they pose a life‐threatening risk. In our case, early ultrasonographic suspicion enabled timely surgical intervention, likely preventing hemorrhage or uterine rupture [1]. Delayed diagnosis is expected due to the anatomical and vascular characteristics of the cornual region, which allow gestational sacs to expand with minimal symptoms [3].

The patient's prior laparotomy for endometriosis and tubal surgeries significantly may have contributed to the ectopic pregnancy. History of laparotomy for endometriosis and tubal resection, hydrosalpinx can impact implantation sites. Our patient had undergone bilateral salpingectomy due to bilateral hydrosalpinx, which altered her tubal—and possibly uterine—anatomy, increasing the risk of cornual ectopic implantation. According to the literature, tubal damage caused by previous ectopic pregnancies, pelvic inflammatory disease, or tubal malformations is a known risk factor for ectopic pregnancies, especially after IVF cycles. In a similar case reported in the literature, a patient with bilateral salpingectomy due to hydrosalpinx and uterine septum resection also experienced a cornual ectopic pregnancy following IVF, highlighting that even after correction of uterine anomalies and removal of fallopian tubes, the risk persists. Therefore, the combination of assisted reproductive technology and her extensive surgical history likely contributed to the development of the cornual ectopic pregnancy in our patient [4]. Other studies have noted the increased risk of cornual pregnancies after ART, with IVF often cited as a risk factor [5, 6]. This highlights the importance of close monitoring and advanced imaging, such as three‐dimensional transvaginal ultrasonography, which confirmed the diagnosis in our patient [6].

Management, in this case, required a combined hysteroscopy and minilaparotomy due to the impending rupture. This aligns with literature recommending surgery when medical management is insufficient, particularly with elevated beta‐HCG levels or high rupture risk [7]. Conservative methotrexate management is possible in early cases with low beta‐HCG and minimal rupture risk [3]. For our patient, surgery was necessary due to the gestational sac size and rupture risk.

Prompt diagnosis and intervention are crucial in managing cornual pregnancies. While our patient avoided rupture, literature reports of ruptured cornual pregnancies often involve significant hemorrhage and emergency surgery [8]. These cases underscore the high mortality rates with delayed diagnosis and the need for early intervention [8].

Careful follow‐up is also essential. Monitoring beta‐HCG levels post‐surgery ensures complete resolution, as shown in our patients and others [6, 7]. Future pregnancies after cornual pregnancies carry a rupture risk, with elective cesarean section around 36–37 weeks recommended to avoid complications [3].

This case highlights the critical need for careful monitoring of ectopic pregnancies, particularly cornual implantations following IVF‐ET, due to the risk of early rupture and severe hemorrhage. Despite a history of prior salpingectomy, ectopic pregnancies can still occur and present significant management challenges.

## 4 Conclusion

This case highlights the diagnostic and management challenges of cornual pregnancies, especially following ART. Early diagnosis, vigilant monitoring, and timely surgical intervention are essential for preventing complications and improving outcomes.

## Author Contributions

**Fatemeh Keikha:** conceptualization, data curation, investigation, project administration, resources, supervision, validation, writing – review and editing. **Hawraa Shbeeb:** conceptualization, data curation, investigation, project administration, resources, supervision, validation, writing – original draft, writing – review and editing. **Huma Homam:** conceptualization, data curation, investigation, resources, validation, writing – original draft, writing – review and editing. **Najmeh Nasiri Khormoji:** investigation, validation, writing – review and editing. **Mahdiye Nouri:** methodology, resources, software, writing – original draft.

## Ethics Statement

We strictly adhered to the principles of the Declaration of Helsinki throughout the whole study process. Also, this study was approved by the Research and Ethics Committee of the Tehran University of Medical Sciences.

## Consent

Written and formal consent for the publication of this case report was obtained from the patient.

## Conflicts of Interest

The authors declare no conflicts of interest.

## Data Availability

The authors have nothing to report.
